# The impact of advanced pre-hospital interventions on scene time

**DOI:** 10.1186/s13049-026-01613-5

**Published:** 2026-04-30

**Authors:** Miles Gandolfi, Flora Bird, Christine L. Henry, Jonathan Bestwick, David J. Lockey, Zane B. Perkins

**Affiliations:** 1 London’s Air Ambulance Charity, The Royal London Hospital, London, E1 1BB UK; 2 Barts Health NHS Trust, London, UK; 3 Imperial College Healthcare NHS Trust, St Mary’s Hospital, London, UK; 4 Wolfson Institute of Population Health, Queen Mary University of London, London, UK; 5 Centre for Trauma Sciences, Queen Mary University of London, London, UK

**Keywords:** Pre-hospital, Emergency, Trauma, HEMS, Procedure, Intervention, Time, Transport, Mechanism, REBOA

## Abstract

**Background:**

Pre-hospital trauma care has evolved with the introduction of increasingly advanced interventions. While these procedures may offer clinical benefit, they may also prolong pre-hospital times, a critical determinant of outcome, particularly in patients with non-compressible haemorrhage. This study examined whether expanding pre-hospital capability has affected scene time over the past two decades.

**Methods:**

We conducted a retrospective, observational study of injured patients treated by a physician-led air ambulance service. Data from July of each year between 2005–2010 (Group 1) and 2017–2021 (Group 2) were compared. Advanced interventions including blood transfusion, Resuscitative Endovascular Balloon Occlusion of the Aorta (REBOA), arterial and central venous cannulation were introduced between these periods. Univariate and multivariable analyses were performed to identify factors associated with scene time.

**Results:**

Among 1,357 eligible patients, 26% received at least one advanced intervention, with uptake increasing over time (24% vs 29%; *p* = 0.02). The proportion of penetrating trauma also increased (24% vs 34%; *p* < 0.001), and these patients had consistently shorter scene times than those with blunt trauma (10 [6–17] vs 25 [17–36] minutes; *p* < 0.001). Median scene time remained similar between study periods. In multivariable analysis, scene time was independently associated with mechanism of injury, age, and number of advanced interventions performed. Each additional intervention increased scene time by 41% (*p* < 0.001).

**Conclusion:**

Advanced pre-hospital interventions have become more frequent, and scene time increases in direct proportion to the number of interventions performed, independent of temporal, operational, patient, and injury factors. Scene times have remained similar over two decades, reflecting opposing trends of increasing intervention frequency and more penetrating trauma. These findings highlight the need to balance the potential benefit of advanced pre-hospital interventions against their time cost, ensuring procedural efficiency, judicious patient selection, and timely transfer to definitive care in time-critical trauma.

**Supplementary Information:**

The online version contains supplementary material available at 10.1186/s13049-026-01613-5.

## Background

Trauma is a leading cause of death globally [1]. In patients with time-critical injuries, outcomes are strongly influenced by the time to definitive treatment [2], whether this is opening an occluded airway, starting a blood transfusion, controlling haemorrhage, or evacuating an intracranial bleed. Delays in access to definitive care have been consistently associated with worse outcomes in trauma, particularly in patients with haemorrhage or traumatic brain injury. Minimising pre-hospital time has long been a core principle of trauma care, supported by consistent evidence linking delays in reaching definitive care with increased mortality [3, 4]. This is particularly true in patients with non-compressible torso haemorrhage or penetrating trauma, where there is a strong correlation between increased scene time and mortality [5, 6].

To improve outcomes, advanced pre-hospital teams have progressively expanded their capability to deliver critical interventions at scene [7–10]. These include pre-hospital emergency anaesthesia, blood transfusion, advanced vascular access, and in selected cases, procedures such as resuscitative thoracotomy or REBOA [11–16]. The aim of these interventions is to prevent pre-hospital death or delay deterioration until definitive in-hospital intervention can be delivered since many definitive treatments, such as surgical haemorrhage control or evacuation of intracranial haematomas, remain available only in hospital. This creates a clinical and operational tension: earlier access to some therapies may delay access to others [17, 18].

This study examined whether the increasing availability and use of advanced pre-hospital interventions has influenced pre-hospital time, and therefore time to definitive care, in a large urban trauma system. We analysed trends over a 16-year period using data from a physician-led trauma service and evaluated the contribution of patient, injury, and intervention factors to pre-hospital time. The aim was to determine whether expanding pre-hospital capability has affected scene time and, ultimately, timely access to definitive care.

## Methods

### Study design

This is a retrospective cohort study of injured patients treated by London’s Air Ambulance (LAA). Following institutional review, this study was classified as a service evaluation, waiving the need for full ethics committee oversight in alignment with UK Health Research Authority guidance. It was registered with the Clinical Effectiveness Unit at Barts NHS Trust, London, UK (reference number 12855). The study adheres to the Strengthening the Reporting of Observational Studies in Epidemiology (STROBE) guidelines.

### Setting

LAA is a physician-paramedic pre-hospital trauma service operating across Greater London, serving a predominantly urban population of approximately ten million. It forms part of the London Trauma System and responds to incidents of major trauma in support of the London Ambulance Service (LAS). During the study period, LAA operated a single team available 24 h a day, by helicopter or rapid response car [19]. The service typically attends 1800 to 2000 patients annually, with a case mix focussed on high-acuity trauma [20]. The tasking is performed by a separate LAA paramedic working in the LAS control room who screens incoming calls and dispatches the team to cases of suspected major trauma based on information recorded by the emergency call taker and on listening into the incoming call. Paramedics working for LAA work interchangeably between the dispatch role and the response role.

Each responding team is comprised of a senior doctor and a paramedic, with a third clinician (doctor or paramedic) increasingly included in later years. When necessary, the team can perform advanced on-scene interventions, including pre-hospital anaesthesia, blood transfusion, resuscitative balloon occlusion of the aorta (REBOA), and resuscitative thoracotomy.

### Study population

The study included injured patients treated by LAA during the month of July in two distinct time periods: 2005–2010 (Group 1) and 2017–2021 (Group 2). July was selected as a pragmatic and consistent sampling frame across study years, allowing case-level data extraction over extended periods while remaining feasible. Exploratory analysis confirmed that July was broadly representative of annual activity (additional file 1). Four advanced interventions: blood transfusion; Resuscitative Endovascular Balloon Occlusion of the Aorta (REBOA); arterial cannulation and central venous cannulation were introduced between 2010 and 2017, meaning data was not collected between these two dates to give two groups to compare. Only primary missions, defined as pre-hospital response directly to the scene of injury, were included. Missions were excluded if the team was stood down before reaching the patient, if the patient was not traumatically injured, if scene time could not be determined due to missing data or if the patient was declared dead at scene. Patients declared dead at scene were excluded, as scene time in this group does not reflect the clinical care processes under study.

### Data collection

Data are collected prospectively on all LAA patients. This includes contemporaneous completion of a patient report form and electronic database including all time stamps of the mission. These are audited monthly to ensure accuracy. We retrospectively collected the required data from patient records held on the pre-hospital electronic patient database (OnBase, Hyland Software Inc., OH, USA). Data were collected on patient demographics, mechanism of injury, interventions performed, team size, mode of transport and all pre-hospital times. Mechanism of injury was categorised as blunt trauma or penetrating trauma. Other mechanisms (drowning, electrocution, burns, and medical patients) were excluded from this study.

Pre-hospital interventions were categorised as regular interventions and advanced interventions for analysis. Advanced interventions include the following eight procedures: Blood transfusion, pre-hospital emergency anaesthesia (PHEA), central venous cannulation, arterial cannulation, thoracostomy, thoracotomy, REBOA and advanced life support for patients in traumatic cardiac arrest.

Pre-hospital time was divided and defined as follows: Injury to activation is the time from emergency call to the HEMS team being activated; activation to on-scene time is the time from HEMS team activation to arrival on scene; scene time is defined as time from HEMS team arrival at the patient to the team’s departure from scene; Transport time is time from leaving scene to arrival at hospital. Total pre-hospital time is the total time taken from injury to arrival at the receiving hospital. The emergency call time was taken to be a surrogate marker for time of injury.

### Analysis

Statistical analyses were performed using IBM SPSS Statistics (Version 27,IBM Corp, NY) and Stata (version 18, StataCorp, College Station, Texas). Data distribution was assessed on histograms and using the Shapiro–Wilk test. Continuous data are reported as median with interquartile range and categorical data as frequency (n) and percentage. Participant characteristics were compared using the Mann Whitney U or Chi Squared tests as appropriate. Tests were 2-sided and *p* value < 0.05 was considered significant. A univariate and multivariate analysis (MVA) was performed using generalised linear models with a gamma distribution and log link function, with the advanced interventions as a continuous variable, and looking at the contribution to the change in scene time from each variable in both unadjusted and fully adjusted analysis.

## Results

A total of 1,357 trauma patients met the inclusion criteria in the two study periods: 728 in Group 1 (2005–2010) and 629 in Group 2 (2017–2021). Exclusions included 154 patients declared dead at scene, 82 with non-traumatic presentations, and four with missing time data. The median patient age was 30 years (range < 1 to 97), and 1082 (81.8%) were adults (age > 18 years).

**Fig. 1:**
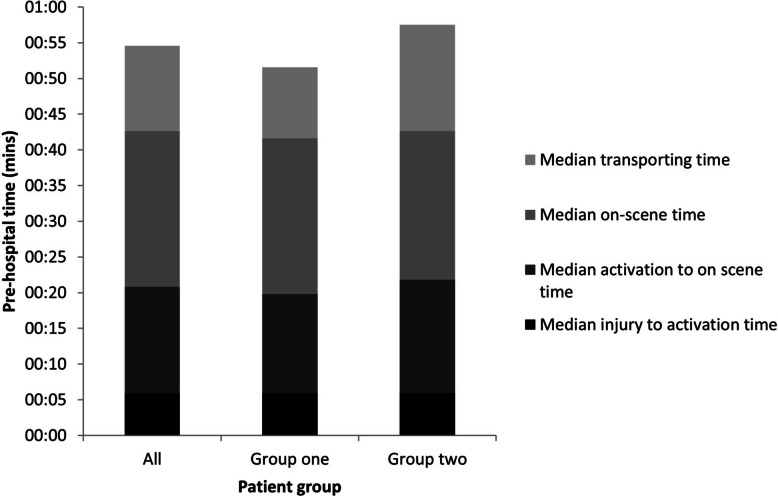
Displays the contribution of individual timing elements of each job to total pre-hospital time in all patients, in group one and in group two

There were important differences in the baseline characteristics of the two cohorts (Table 1). Penetrating trauma was less common in Group 1 compared to Group 2 (176 of 728 (24.2%) versus 215 of 629 (34.2%); *p* < 0.001). Patients in group 1 were also less likely to receive an advanced intervention (172 of 728 (23.6%) versus 185 of 629 (29.4%); *p *= 0.02). The majority of patients in both groups received no advanced interventions. Other significant differences included a higher frequency of three-person teams and a higher proportion of helicopter responses in Group 1. Figure 1 shows total pre-hospital times, displaying the contribution of each component to the total time in all patients, in group one and in group two.

**Table 1:** Baseline characteristics of the study population

Variable	Group 1 (2005–10) n 728	Group 2 (2017–21) n 629	*P*-value
**Age in years** (range)	27 (0–93)	31 (0–97)	** < 0.001**
**Pre-hospital times** (minutes)			
Activation to on scene	21 (16–28)	24 (19–32)	** < 0.001**
Scene time	22 (11–33)	20 (11–31)	0.158
Transport time	10 (6–16)	15 (10–21)	** < 0.001**
Total pre-hospital time	59 (46–76)	63 (51–80)	** < 0.001**
Scene time, no advanced intervention	17 (10–28)	16 (9–23)	** < 0.001**
Scene time with ≥ 1 advanced intervention	36 (28–46)	36 (29–46)	0.857
**Mechanism of injury:**			
Blunt	552 (75.8%)	414 (65.8%)	** < 0.001**
Penetrating	176 (24.2%)	215 (34.2%)	** < 0.001**
**Team size:**			
2 person	501 (68.8%)	263 (41.8%)	** < 0.001**
3 person	227 (31.2%)	366 (58.2%)	** < 0.001**
**Mode of transport to scene:**			
Aircraft	382 (52.5%)	291 (46.3%)	**0.023**
Fast response car	346 (47.5%)	338 (53.7%)	**0.023**
**Advanced intervention:**			
No advanced intervention	556 (76.4%)	444 (70.6%)	**0.015**
≥ 1 advanced intervention	172 (23.6%)	185 (29.4%)	**0.015**
PHEA	155 (21.3%)	154 (24.5%)	0.081
Thoracostomies	35 (4.8%)	38 (6.0%)	0.164
Thoracotomy	10 (1.4%)	3 (0.5%)	**0.047**
Advanced life support*	16 (2.2%)	11 (1.7%)	0.254
Blood transfusion	2 (0.2%)	56 (8.9%)	** < 0.001**
Central venous cannulation	3 (0.4%)	13 (2.1%)	**0.002**
Arterial cannulation	1 (0.1%)	14 (2.2%)	** < 0.001**
REBOA	0 (0%)	3 (0.5%)	

*Abbreviations*: *PHEA* Pre-hospital Emergency Anaesthesia, *REBOA* Resuscitative Balloon Occlusion of the Aorta

^*^ Advanced Life Support signifies the HEMS treatment of a patient in traumatic cardiac arrest, utilising a variety of treatment options that the team felt appropriate such as closed chest compressions and ventilation. Age was not documented in 12 and 23 of cases in group one and group two respectively. Scene time was available for all cases, but total pre-hospital time was not available for 172 and 113 of group one and group two respectively due to time of arrival at hospital not being recorded

*p*<0.05 is formatted in bold to indicate statistical significance

### Mechanism of injury

Differences between blunt and penetrating trauma are summarised in Table 2. Patients with blunt trauma were older and more likely to receive at least one advanced intervention.

**Table 2:** Scene times, demographics and interventions performed in blunt and penetrating trauma in all patients

Variable	Penetrating trauma n 391 (28.8%)	Blunt trauma n 966 (71.2%)	*P*-Value
**Age in years** (interquartile range)	24 (19–35)	31 (21–46)	** < 0.001**
**Pre-hospital times** (minutes)			
Activation to on scene	22 (18–27)	23 (17–31)	0.065
Scene time	10 (6–17)	25 (17–36)	** < 0.001**
Total pre-hospital time	49 (38–61)	67 (54–83)	** < 0.001**
Scene time, no advanced intervention	9 (6–15)	21 (14–29)	** < 0.001**
Scene time with ≥ 1 advanced intervention	28 (17–39)	36 (29–49)	** < 0.001**
**Team size:**			
2 person	225 (57.5%)	539 (55.8%)	0.284
3 person	166 (42.5%)	427 (44.2%)	0.284
**Mode of transport to scene:**			
Aircraft	163 (41.7%)	521 (53.9%)	** < 0.001**
Fast response car	228 (58.3%)	445 (46.1%)	** < 0.001**
**Advanced intervention:**			
No advanced intervention	340 (87.0%)	660 (68.3%)	** < 0.001**
≥ 1 advanced intervention	51 (13.0%)	306 (31.7%)	** < 0.001**
PHEA	29 (7.4%)	280 (29.0%)	** < 0.001**
Thoracostomies	12 (3.1%)	61 (6.3%)	**0.009**
Thoracotomy	12 (3.1%)	1 (0.1%)	** < 0.001**
Advanced life support*	9 (2.3%)	18 (1.9%)	0.317
Blood transfusion	20 (5.1%)	38 (3.9%)	0.160
Central venous cannulation	6 (1.5%)	10 (1.0%)	0.216
Arterial cannulation	3 (0.8%)	12 (1.2%)	0.260
REBOA	0 (0%)	3 (0.3%)	

*Abbreviations*: *PHEA* Pre-hospital Emergency Anaesthesia, *REBOA* Resuscitative Balloon Occlusion of the Aorta

^*^ Advanced Life Support signifies the HEMS treatment of a patient in traumatic cardiac arrest, utilising a variety of treatment options that the team felt appropriate such as closed chest compressions and ventilation. Age was not documented in 9 and 26 cases in the penetrating and blunt trauma groups respectively. Scene time was available for all cases, but total pre-hospital time was not available for 56 and 229 of the penetrating and blunt trauma groups respectively due to time of arrival at hospital not being recorded

*p*<0.05 is formatted in bold to indicate statistical significance

**Fig. 2:**
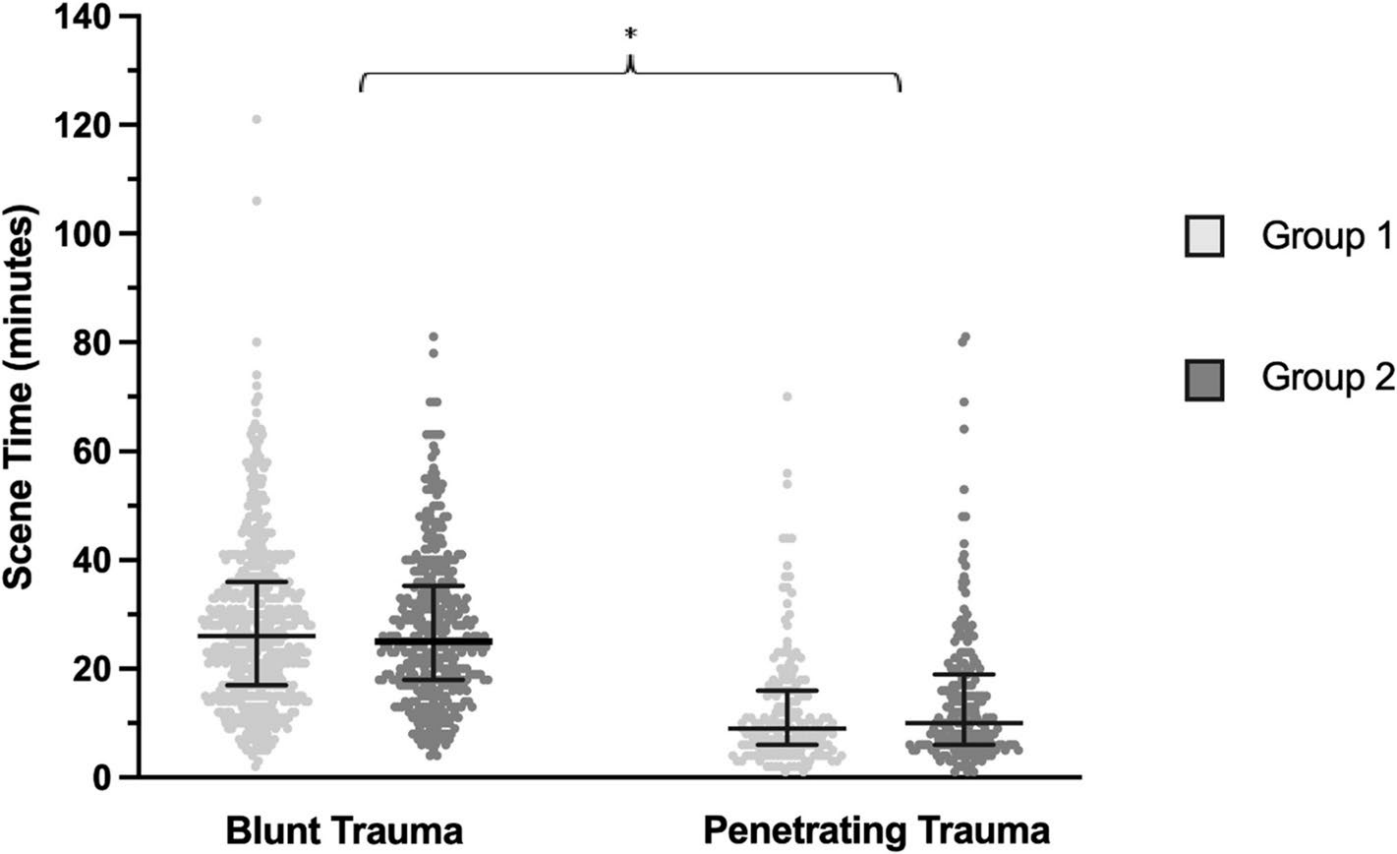
Compares groups one and two with regards to scene time for blunt and penetrating injury. Each mission is displayed, with median and interquartile range marked. * Notates the significant difference between the scene times in blunt and penetrating groups in both groups one and two

Scene time differed significantly by mechanism of injury. Median scene time was longer for blunt trauma than for penetrating trauma (25 [IQR 17–36] vs 10 [IQR 6–17] minutes; *p* < 0.001), a 2.5-fold difference that persisted across both study periods (Group 1: 26 (IQR 17–36) vs 9 (IQR 6–16) minutes, *p* < 0.001; Group 2: 25 (IQR 18–35) vs 10 (IQR 6–19) minutes, *p* < 0.001) (Fig. 2). The proportion of penetrating trauma patients receiving > 1 advanced intervention was unchanged between periods (7.4% vs 5.0%, *p *= 0.899), whereas among blunt trauma patients it more than doubled (4.9% to 11.6%, *p *< 0.01).

### Interventions

**Fig. 3:**
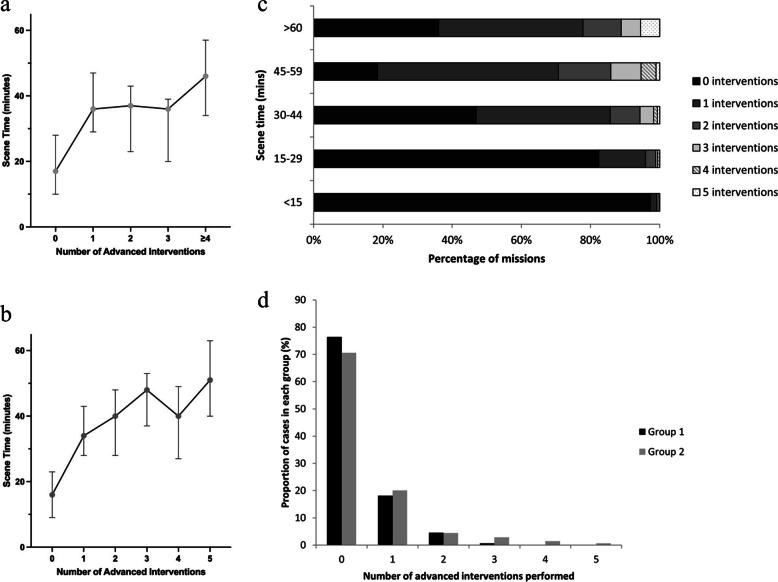
**a**-**d** Separate patients into groups based on how many advanced interventions they received. **a** and **b** Median scene time for patients grouped by number of advanced interventions received split into groups one (**a**) and two (**b**), with error bars signifying IQR. Line is solely to show trend of increasing scene time with increased number of interventions performed. x axis of 3a ends at > 4, compared to the x axis of 3b ending at 5 as only two patients in group one had more than 3 interventions, compared to 13 in group two. **c** Percentage of jobs within certain time points which received a specific number of interventions. **d** Proportion of cases within groups one and two with certain numbers of advanced interventions performed

Most patients received no advanced intervention, but where these were performed, they were associated with longer scene times (Fig. 3a, b). Median scene time in patients receiving no advanced intervention was 17 (IQR 10–25) minutes, increasing to 57 (IQR 40–61) minutes in those who received five interventions. There was a clear trend towards longer scene times with increasing intervention burden (Fig. 3c).

Analysis of median scene times for missions including each advanced intervention was undertaken (Additional File 2) with pre-hospital emergency anaesthesia (PHEA) being the most frequently undertaken advanced intervention, performed in 309 patients (22.8%). Median scene time in this group was 37 (IQR 30–47) minutes, with no difference between Group 1 and Group 2 (36 (IQR 29–48) vs 38 (IQR 30–47) minutes; *p* = 0.960).

The maximum number of advanced interventions performed on a single patient was five, observed in both study periods. As shown in Fig. 3d, a greater proportion of patients in Group 1 received no advanced interventions, whereas Group 2 saw more cases involving three or more interventions. Only two patients in Group 1 had more than three advanced interventions, while 13 patients in Group 2 had more than three interventions. Among individual procedures, REBOA was associated with the longest median scene time (61 [IQR 56–65] minutes).

### Multivariable analysis

In adjusted analysis (Table 3), the number of advanced interventions was a strong independent predictor of scene time. Each additional intervention was associated with a 41% increase in scene time (relative change 1.41; 95% CI 1.35–1.48; *p* < 0.001). Mechanism of injury was also an independent predictor: scene time for blunt trauma was almost twice that for penetrating trauma (relative change 1.91; 95% CI 1.78–2.05; *p* < 0.001).

**Table 3:** Multivariate analysis looking at the relative change in scene time contributed to by different variables

	**Relative change in mean scene time**	
	**Unadjusted**	**Adjusted**
**Group:**		
2005–2010	1 (ref)	1 (ref)
2017–2021	0.949 (0.885 to 1.017); *p* = 0.136)	0.939 (0.878 to 1.003); *p* = 0.062
**Age (per year)**	1.006 (1.004 to 1.008); *p* < 0.001	1.002 (1.000 to 1.004); *p* = 0.013
**Activation to on-scene (per minute)**	1.004 (1.001 to 1.007); *p* = 0.005	1.002 (0.999 to 1.004); *p* = 0.201
**Mode of transport**		
Car	1 (ref)	1 (ref)
Aircraft	0.839 (0.783 to 0.898); *p *< 0.001	0.895 (0.840 to 0.953); *p* = 0.001
**Clinical team size:**		
2 person	1 (ref)	1 (ref)
3 person	1.066 (0.994 to 1.143); *p *= 0.073	1.030 (0.963 to 1.100); *p* = 0.389
**Advanced interventions (continuous)**	1.461 (1.393 to 1.533); *p* < 0.001	1.409 (1.347 to 1.475); *p *< 0.001
**Penetrating trauma:**		
No	1 (ref)	1 (ref)
Yes	0.487 (0.451 to 0.526); *p* < 0.001	0.524 (0.488 to 0.563); *p* < 0.001
**Weekend:**		
No	1 (ref)	1 (ref)
Yes	0.915 (0.853 to 0.982); *p* = 0.013	0.953 (0.894 to 1.017);* p* = 0.146
**Night:**		
No	1 (ref)	1 (ref)
Yes	0.961 (0.878 to 1.052); *p* = 0.390	1.026 (0.945 to 1.114); *p* = 0.542

Weekend = Friday 8pm-Monday 8am, Night = 8pm to 8am

Age was not documented in 35 cases

A clinically small but statistically significant association was seen with age, with a 0.2% increase in scene time for every year increase in age (*p* < 0.001). After adjusting for these variables, there was no significant difference in scene time between the two study periods (*p* = 0.062), supporting the observation that changes in pre-hospital time over the years are better explained by shifts in case mix and intervention burden than by temporal factors alone.

## Discussion

This study found that average scene times have remained similar over the past two decades despite the increasing use of advanced pre-hospital interventions. Although more procedures were undertaken in later years, with the introduction of four interventions into routine use, a higher proportion of penetrating trauma – characterised by significantly shorter scene times – and a continued majority of patients receiving no advanced intervention likely account for the overall unchanged scene times. Among individual patients, however, scene time increased progressively with each additional advanced intervention. After adjustment, the independent predictors of longer scene time were mechanism of injury, number of advanced interventions, age, and mode of transport to scene; calendar period had no independent effect.

Mechanism of injury had the strongest association: scene time for blunt trauma was almost twice that for penetrating trauma. This may reflect both the greater procedural requirements of blunt trauma and the long-standing operational principle within the service of maintaining deliberately short scene times for penetrating injuries – treated as an ‘intervention’ in its own right. This approach derives from extensive experience managing penetrating trauma, often associated with non-compressible haemorrhage, for which minimising pre-hospital time is reported to improve survival [5, 6].

Each additional advanced intervention increased scene time by 41%. The authors recognise that some procedures are inherently more time consuming than others, and some may occur concurrently, which limits direct comparisons between individual interventions. Individual analysis of scene time for each advanced procedure is provided (Additional File 2) but should be interpreted with caution. Pre-hospital care is complex, and interventions are performed in parallel rather than sequentially. As such, individual procedures cannot be considered independent exposures. In addition, there is substantial confounding by indication: patients requiring specific interventions are typically more severely injured and require more complex care, which itself prolongs scene time. Any estimate of additional scene time from a given intervention therefore reflects both the procedure and the underlying clinical context.

Although patient outcomes were not included in this study, previous studies have demonstrated strong associations between longer pre-hospital times and mortality, particularly in penetrating trauma and non-compressible haemorrhage [2–6]. This study does not suggest that all advanced interventions should be eschewed in order to solely focus on scene time, rather that nuanced decision-making is utilised in determining when to proceed with an intervention and when to refrain. Recent improvements in survival from pre-hospital traumatic cardiac arrest suggest that an appropriate balance can be achieved between rapid transport and the delivery of potentially life-saving interventions [18].

The modest effect of age on scene time (0.2% per year) was unlikely to be clinically meaningful and may reflect confounding by mechanism, as older patients were more likely to sustain blunt trauma. The association between helicopter response and shorter scene times was unexpected. This may reflect a confounding effect of daylight operations, during which scenes are typically better resourced and supported by ambulance service personnel, rather than any intrinsic advantage of aircraft deployment. However, no data were available to test this hypothesis.

The increase in total pre-hospital time between the two periods was driven by longer activation-to-scene and transport intervals. The latter may relate to the 2010 introduction of London's Major Trauma System, which directed these patients to designated Major Trauma Centres, formalising the bypass of local hospitals, increasing the frequency of these potentially longer journeys. The longer activation-to-scene interval (by approximately three minutes) could reflect a change in case mix with a higher proportion of penetrating trauma. Penetrating trauma cases often require interrogation before dispatch, whereas a number of blunt mechanisms trigger an automatic dispatch of the HEMS team [19]. Given the time-critical nature of this group, even small delays are potentially important [21], and future work should explore modifiable contributors to response time.

The rise in penetrating trauma and decline in blunt trauma likely reflect broader changes in urban injury patterns, including improved road safety and a sustained increase in knife-related violence [22, 23]. Greater use of advanced interventions may reflect expanded capability or improved tasking, rather than higher patient acuity, which is supported by internal and published data showing stable injury severity over time [19, 24, 25]. The increase occurred predominantly in blunt trauma patients, with no change in intervention frequency among those with penetrating injury. Scene times for this urban trauma service were comparable with other regional and international pre-hospital systems [26–29].

We acknowledge that this study does not report patient-centred outcomes such as mortality or functional status. However, time to definitive care is a well-established determinant of outcome in trauma, particularly in haemorrhage and traumatic brain injury [4, 5, 30]. In this context, scene time represents a clinically meaningful process measure. Our findings demonstrate that increasing pre-hospital intervention burden is independently associated with longer scene times, providing empirical data to inform a key operational trade-off in trauma care: the balance between delivering early interventions and expediting transfer to definitive treatment.

This study has several limitations. Its retrospective design introduces potential selection and measurement bias, though contemporaneous data recording and audit reduce this risk. There are also the risks of collider bias and of systematic measurement error of prehospital time, which along with potential limitations of the internal validity of the multivariable model and the risk of residual confounding, which may exist from unmeasured variables, may bias this data. The recorded data was of high quality, with only four patients (0.2%) excluded due to missing information. Missing hospital-arrival time stamps limited calculation of total pre-hospital time in some cases, but scene time data were complete. Using data from the month of July may not be fully representative of the service, as there may be some seasonal variation in case mix. Patients who died on scene were excluded as they were not taken for definitive care, but patients in this group received advanced interventions, which would have increased their scene time and increased their risk of mortality. The data collection did not include patient injuries or injury severity scores, limiting the external transferability to other services. However, we know from previous studies of this service that the median Injury Severity Score is 17 [24]. When assessing the number of advanced interventions performed against scene time, it is not possible to distinguish which, if any, were initiated en-route to hospital as opposed to on-scene. However, during the study period it was the service’s practice that the vast majority of interventions be performed or initiated on scene. We appreciate the limited external validity when comparing this data to other HEMS services, as LAA is primarily focused on trauma in a highly urban environment. However, it offers insight into the pre-hospital times, types of injury and interventions utilised in this setting.

## Conclusion

While the availability and uptake of advanced pre-hospital interventions has increased over the past two decades, overall scene times have not changed. However, this is likely explained by a shift toward more penetrating trauma and a continued majority of patients receiving no advanced interventions. Among patients who do receive advanced interventions, scene times increase with each additional procedure, highlighting the importance of operational efficiency and careful triage in deciding when these time-costly interventions are appropriate. We suggest further work is needed to define which patients would benefit most from these advanced pre-hospital interventions.

## Supplementary Information

Additional file 1: Figures a and b—July is broadly representative of annual activity with regard to numbers of patients seen per month in both Group 1 (Figure a) and Group 2 (Figure b), with the month of July highlighted in red. Figures c and d—July is broadly representative of annual activity with regard to mechanism of injury in both Group 1 (Figure c) and Group 2 (Figure d), with the month of July denoted by month 7.

Additional file 2: Unadjusted median scene times for each advanced intervention analysed in this project, and for missions with no interventions carried out are displayed. Interventions not carried out in isolation, and each median scene time is not adjusted for other interventions carried out on the same mission.

## Data Availability

The datasets used and/or analysed during the current study are available from the corresponding author on reasonable request.

## Abbreviations

LAA: London’s air ambulance
HEMS: Helicopter Emergency Medical Service
PHEA: Pre-hospital emergency anaesthesia
REBOA: Resuscitative endovascular balloon occlusion of the aorta
MVA: Multivariate analysis
STROBE: Strengthening the Reporting of Observational Studies in Epidemiology
