# Supplementary material for: The impact of advanced pre-hospital interventions on scene time
Source: Scand J Trauma Resusc Emerg Med. 2026 Apr 30;34:108. doi: 10.1186/s13049-026-01613-5 (PMC13277237; doi:10.1186/s13049-026-01613-5)
Supplement: Supplementary file 1 — Additional file 1: Figures a and b—July is broadly representative of annual activity with regard to numbers of patients seen per month in both Group 1 (Figure a) and Group 2 (Figure b), with the month of July highlighted in red. Figures c and d—July is broadly representative of annual activity with regard to mechanism of injury in both Group 1 (Figure c) and Group 2 (Figure d), with the month of July denoted by month 7. [file 13049_2026_1613_MOESM1_ESM.pptx]

## Slide 1
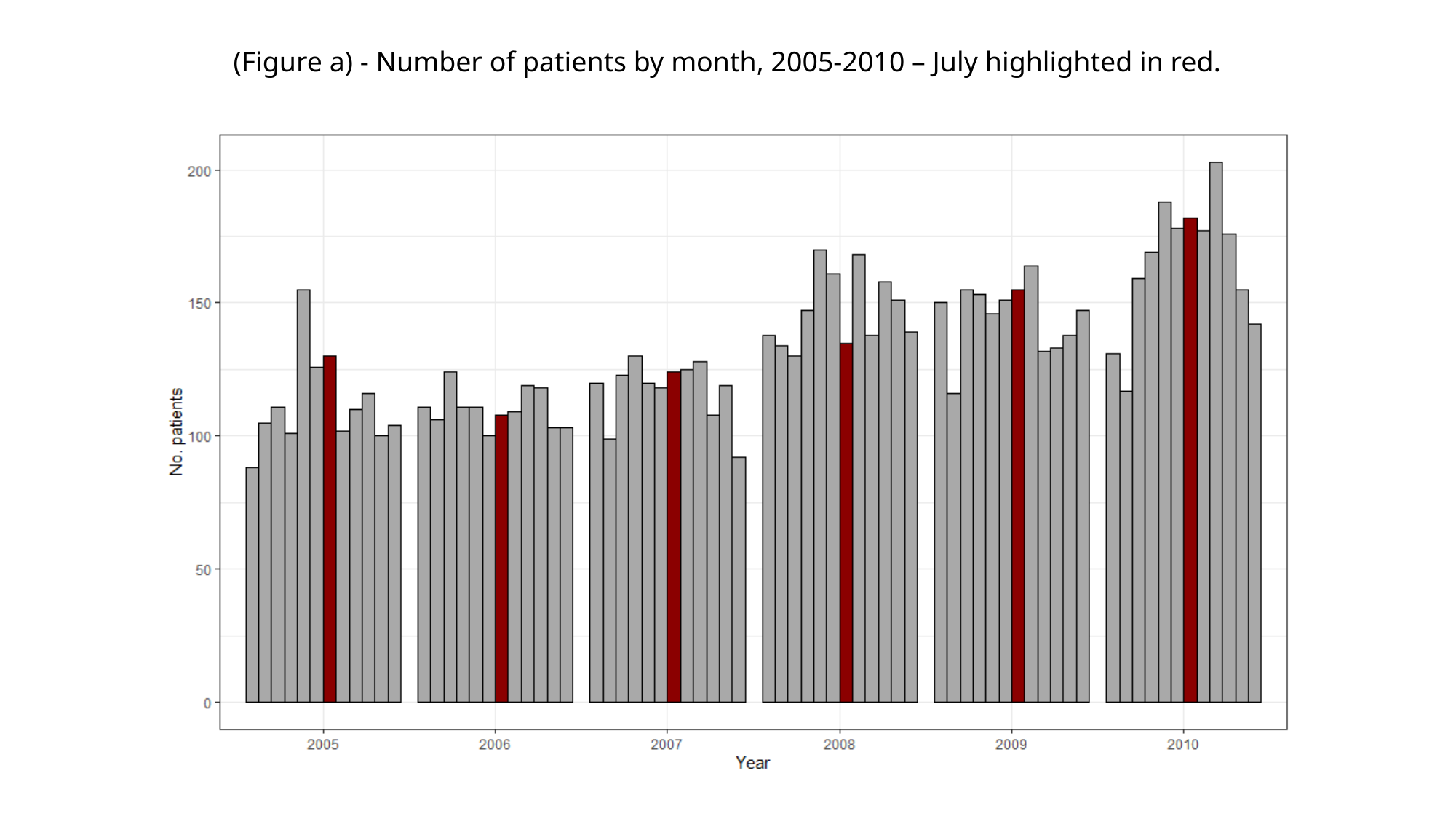

(Figure a) - Number of patients by month, 2005-2010 – July highlighted in red.

## Slide 2
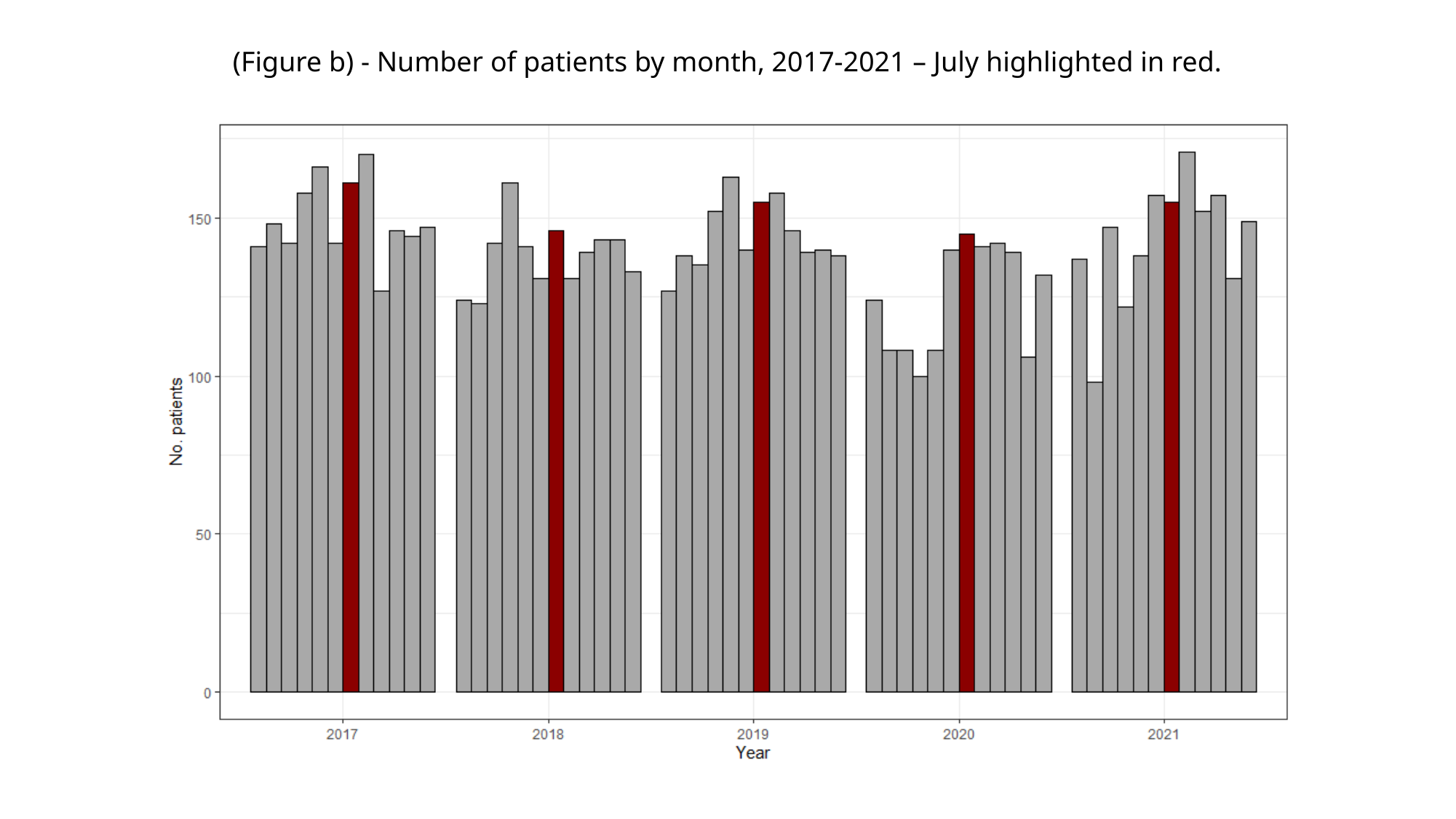

(Figure b) - Number of patients by month, 2017-2021 – July highlighted in red.

## Slide 3
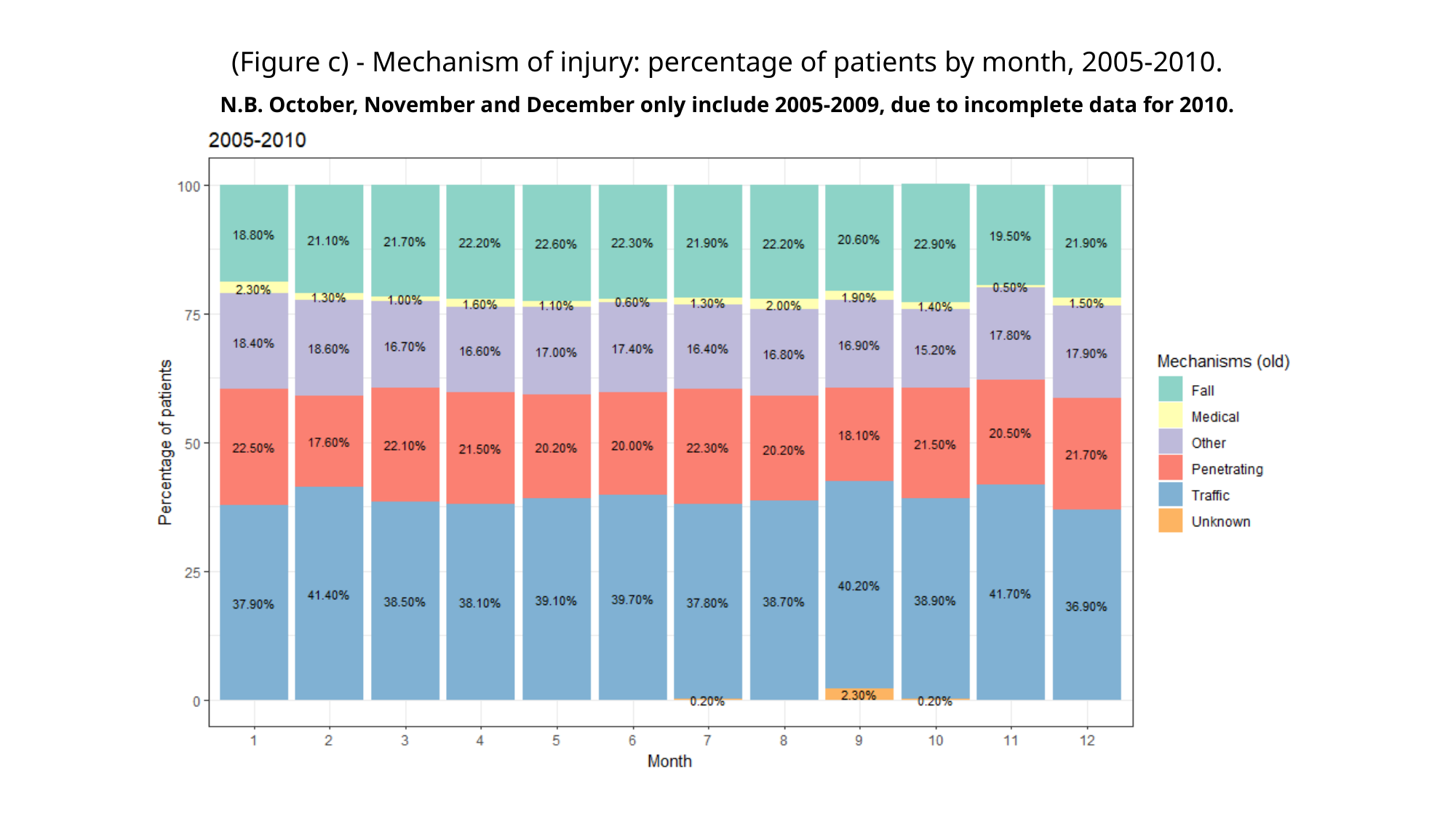

(Figure c) - Mechanism of injury: percentage of patients by month, 2005-2010.
N.B. October, November and December only include 2005-2009, due to incomplete data for 2010.

## Slide 4
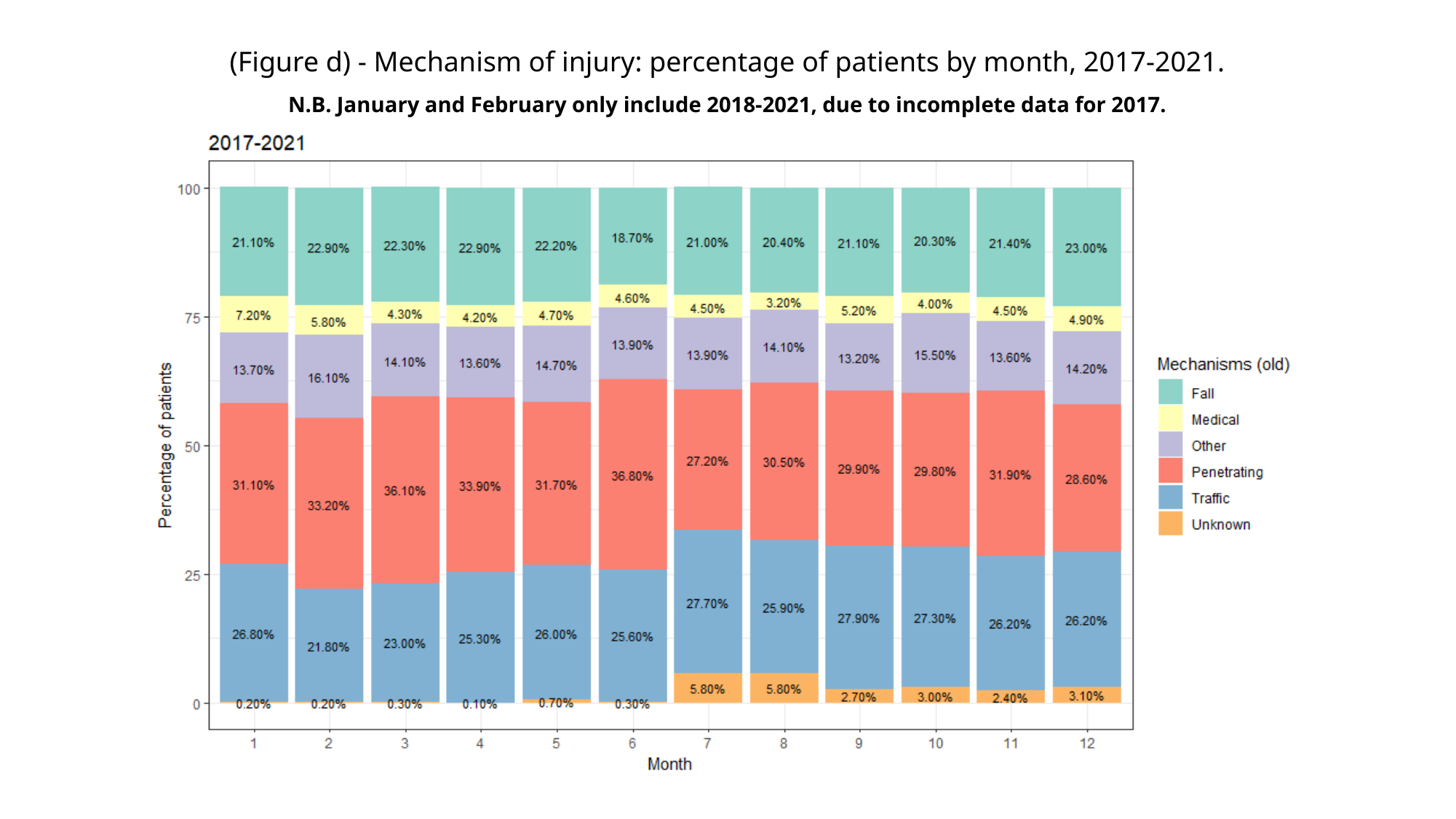

(Figure d) - Mechanism of injury: percentage of patients by month, 2017-2021.
N.B. January and February only include 2018-2021, due to incomplete data for 2017.
