# Supplementary material for: The impact of advanced pre-hospital interventions on scene time
Source: Scand J Trauma Resusc Emerg Med. 2026 Apr 30;34:108. doi: 10.1186/s13049-026-01613-5 (PMC13277237; doi:10.1186/s13049-026-01613-5)
Supplement: Supplementary file 2 — Additional file 2: Unadjusted median scene times for each advanced intervention analysed in this project, and for missions with no interventions carried out are displayed. Interventions not carried out in isolation, and each median scene time is not adjusted for other interventions carried out on the same mission. [file 13049_2026_1613_MOESM2_ESM.docx]

| **Advanced intervention (n)** | **Median scene time in minutes (interquartile range)** |
| --- | --- |
| **None** (1000) | 17 (10-25) |
| **PHEA** (309) | 37 (30-47) |
| **Thoracostomies** (73) | 40 (32-48) |
| **Thoracotomy** (13) | 30 (19-40) |
| **Advanced life support*** (27) | 26 (18-40) |
| **Blood transfusion** (58) | 36 (23-47) |
| **Central venous cannulation** (16) | 40 (40-45) |
| **Arterial cannulation** (15) | 48 (38-54) |
| **REBOA** (3) | 61 (56-65) |
| Abbreviations: PHEA: Pre-hospital Emergency Anaesthesia; REBOA: Resuscitative Balloon Occlusion of the Aorta. * Advanced Life Support signifies the HEMS treatment of a patient in traumatic cardiac arrest, utilising a variety of treatment options that the team felt appropriate such as closed chest compressions and ventilation. Table shows unadjusted median scene time in minutes for all missions for each intervention. Interquartile range is given in brackets. | |

Additional file 2. Table showing unadjusted scene times for each advanced intervention, and those with no advanced interventions carried out.
